# Sudden Unexpected Death in a Patient with Tumour Associated Pulmonary Embolism

**DOI:** 10.1155/2014/396832

**Published:** 2014-11-27

**Authors:** D. Laohachewin, F. André, D. Tschaharganeh, H. A. Katus, G. Korosoglou

**Affiliations:** ^1^Department of Internal Medicine III, Cardiology, Angiology, and Pulmonology, University Hospital of Heidelberg, Im Neuenheimer Feld 410, 69120 Heidelberg, Germany; ^2^Department of Cancer Biology and Genetics, Memorial Sloan Kettering Cancer Center, 1275 York Avenue, New York, NY 10065, USA

## Abstract

Tumour embolisms are rare and in most cases sudden causes of death. Diagnosing this rare condition is still very challenging in the daily clinical routine. In this report we present a case of a lethal sudden pulmonary tumour embolism in a 71-year-old male patient, who was admitted for elective invasive coronary angiography due to suspected coronary artery disease. The patient had suspected Ormond's disease and no previous history of tumour burden. Possible diagnostic and treatment options are discussed herein and an overview of the current literature is also presented.

## 1. Introduction

Pulmonary tumour embolisms are a rare and in most cases sudden cause of death. First described in 1897 by Schmidt [1], many patients present with unspecific symptoms and unknown primary tumours. So far, only reviews of case series have been reported, and a large percentage of such patients end with lethal complications. The pathophysiology is currently not entirely understood [2]. In this regard, rapid and effective diagnostic and appropriate treatment strategies are a challenge for clinicians who treat such patients. In recent review articles, several diagnostic algorithms have been discussed. Such algorithms, however, have been developed for the diagnostic work-up of thrombotic embolisms and are therefore not always helpful in patients with carcinoma associated embolisms [2, 3]. In this context, it is not surprising that fibrinolytic therapy has been unsuccessful in such cases [4]. Herein, we present a case of tumour associated embolism in a patient with suspected Ormond's disease.

## 2. Case Presentation

**Figure 1:**
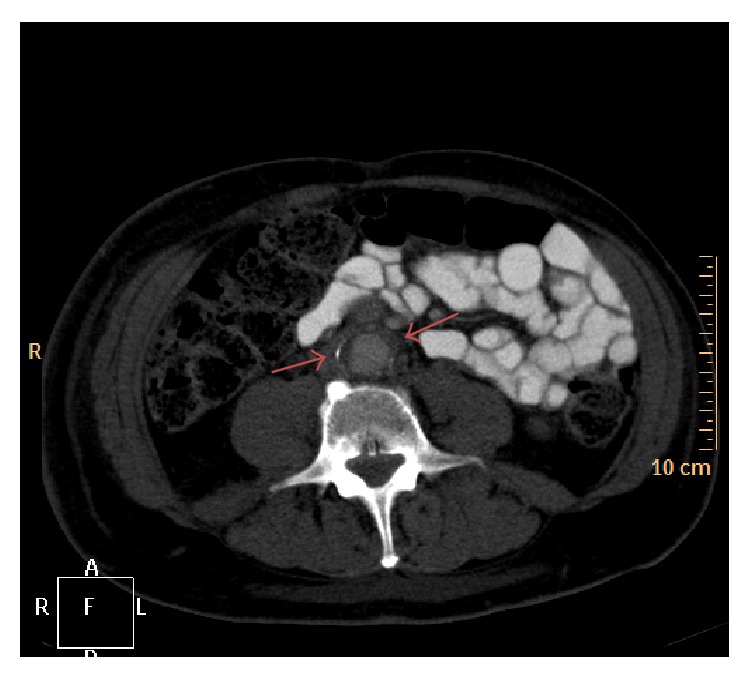
Abdominal CT scan illustrating fibrotic, possibly infiltrative changes around the abdominal aorta (red arrows).

**Figure 2:**
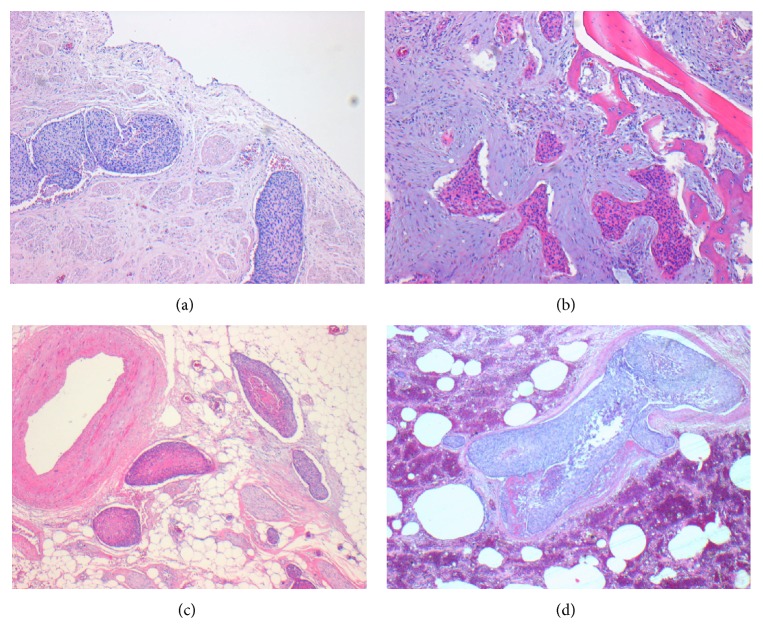
(a) Squamous cell carcinoma manifestation in the ureteral wall. (b) Metastatic manifestation of the squamous cell carcinoma lesion in the vertebral body. (c) Tumour infiltration of periurethral fat tissue. (d) Pulmonary vessels with extensive squamous cell carcinoma tumour embolisms and haemorrhagic infarction of the pulmonary parenchyma.

A 71-year-old patient was admitted to our department for elective coronary angiography due to suspected coronary artery disease. His main complaint was typical angina at moderate exertion (CCS class II). Additionally, he reported swelling in the scrotum and his right leg during the past 6 months. A deep vein thrombosis was ruled out by negative Doppler sonography. A computed tomography (CT) scan on the other hand showed diffuse proliferation of fibrous tissue in the retroperitoneum, compatible with Ormond's disease (Figure 1). Endoscopic evaluation demonstrated mild gastritis and duodenitis, without further malignant findings. Transthoracic echocardiography revealed preserved left ventricular function and a borderline increase of systolic pulmonary arterial pressure of 35 mmHg. Coronary angiography on the other hand revealed a 2-vessel coronary artery disease, with a 50% stenosis of the left main and mid right coronary artery. In this regard, absence of inducible myocardial ischemia was observed using cardiac dobutamine stress magnetic resonance tomography, and therefore PCI was not performed. In addition, a mild dilatation of the RV was noticed with baseline CMR images. During his hospital stay, however, the patient was found unconscious, centralized, and with apnoea, requiring immediate cardiopulmonary resuscitation and admission to our intensive care unit. Echocardiographic examinations revealed akinetic ventricles without pericardial effusion and ECG showed pulseless electrical activity. The patient expired despite prolonged resuscitation. Postmortem autopsy revealed an unexpected diagnosis. Thus, histopathologic work-up showed a squamous cell carcinoma of the right ureter (apT4, apN3, apM1, V1, G2), which infiltrated the pelvis, corresponding vessels, and periurethral tissue and caused metastatic lesions in the vertebral body (Figures 2(a)–2(c)). In addition, massive embolization of cell conglomerates was observed in the pulmonary vessels causing haemorrhagic infarction of the pulmonary parenchyma (Figure 2(d)).

## 3. Discussion

Pulmonary tumour embolisms remain a rare and in most cases sudden cause of death. Unspecific presentation in symptoms of usually previously unknown primary tumours can prevent both diagnosis and effective treatment [2]. Possible hypotheses explaining massive tumour cell embolization include infiltration of the veins with tumour cells, which subsequently enter the circulation causing embolism of the pulmonary arteries. Once such neoplastic cells have entered the pulmonary circulation, they may additionally induce the activation of the coagulation cascade. Therefore, an admixture of thrombus and tumour cells is usually present in such cases. In recent autopsy series, breast and liver tumours were associated with intravascular tumour, while cases of stomach, prostate, and lung carcinoma demonstrated such a mixed pattern [5]. In addition to such intravascular obstructive mechanisms, mural changes resulting in pulmonary hypertension have also been described. Such mechanisms include medial hypertrophy of small arteries and arterioles, fibrointimal proliferation, intimal fibrosis, and disruption of the internal elastic lamina with endarteritis [5]. In contrast to the classical thromboembolic disease, such proliferative and fibrotic changes usually lead to complete and irreversible occlusion [2, 3, 5]. This may explain why “cor pulmonale” tends to develop earlier and right heart failure symptoms tend to be more severe with tumour embolism [4–6].  Table 1 gives an overview of so far reported tumour-related cases of pulmonary embolism (see references listed separately in Supplementary Material available online at http://dx.doi.org/10.1155/2014/396832).

Of course the diagnostic work-up of such cases is challenging for the clinician, since no blood tests or imaging modalities are available for diagnosis. Thus, conventional clinical probability scores [7] would have computed a score of 0 in our case, indicating a very low risk for pulmonary embolism. In addition, standardized blood tests such as measurement of D-dimers [8] probably have low positive and negative predictive value, because baseline tumour activity may lead to false positive and the absence of thrombotic debris within the emboli to false negative results [4]. Plain chest radiographs may be diagnostic in some cases if parenchymal abnormalities, such as fibrotic changes of the lungs, are present [2]. In a case series by Kane et al., however, only one of eight patients has exhibited such parenchymal abnormalities [9]. Computer tomography, on the other hand, has been used in only one study, where “multifocal dilatation and beading of peripheral pulmonary arteries” has been described in 3 cases of pulmonary tumour embolisms [10]. The utility of ventilation/perfusion scans to detect symmetric and peripherally localized defects has also been described [11–13]. Pulmonary angiography, which is still considered as the invasive reference standard technique for the diagnosis of thrombotic embolism, exhibits low sensitivity for the detection of tumour embolisms [14, 15]. Aspiration cytology has also been discussed as a diagnostic alternative but may be difficult to implement in most cases [16, 17].

From a therapeutic point of view, fibrinolytic treatment seems ineffective. One case report has attempted this approach, without ultimate success [4]. As presented in Table 1, some cases, notably atrial myxoma and kidney tumours, have survived due to immediate emergency surgery, which may be a promising alternative in haemodynamic stable patients.

In our case, we followed standard procedures of cardiopulmonary resuscitation [18]. However, our patient unfortunately expired before any diagnosis could be made. Retrospective review of echocardiographic and CMR findings of a borderline increase in pulmonary arterial pressure and mild RV dilatation may had been indicative of previous subclinical embolic events. Even though the definitive diagnosis of our case was set postmortem, having been aware of such pathologic findings beforehand would not have much changed our strategy. An interesting aspect of our case was the additional finding of retroperitoneal fibrotic changes on CT indicative of Ormond's disease. However, retrospective histopathologic work-up showed that these changes were probably related to infiltrative activity of the squamous cell carcinoma, which infiltrated the ureteral wall, periurethral tissue, and probably also the abdominal aorta.

Pulmonary tumour embolisms continue to be clinically challenging in modern medicine. Patient presentation is unspecific and diagnostic options and therapy possibilities remain limited. Further studies are required to establish effective detection and treatment strategies.

## Supplementary Material

Overview of reported cases and their references.

## Figures and Tables

**Table 1:** Overview of reported pulmonary tumour embolisms.

Malignancy	*n*	Chief complaint	*n*	In-hospital mortality	Diagnostic methods used	*n*
Atrial myxoma	9	Chest pain Cough Dyspnea Fever Shock	1 1 7 1 1	33,30%	Autopsy CT Surgery PET/CT	2 6 6 1

Bladder	4	Chest pain Dyspnea None	1 2 1	100%	Autopsy Pulmonary angiogram	4 1

Breast	30	Chest pain Dyspnea None	2 28 2	93,30%	Autopsy Pulmonary angiogram Aspiration cytology Radio nuclear perfusion Surgery Ventilation/perfusion scan	27 4 1 3 1 3

Colon	5	Abdominal pain Cough Dyspnea Fever None	1 1 2 1 1	100%	Autopsy	5

Kidney	10	Chest pain Dyspnea None Shock	1 8 1 1	30%	Autopsy CT Surgery	1 8 8

Liver	9	Chest pain Dyspnea Fever None	2 5 1 2	88,90%	Autopsy Pulmonary angiogram CT Aspiration cytology	8 2 2 1

Lung	12	Cough Dyspnea None Shock	1 9 1 1	91,70%	Autopsy Pulmonary angiogram CT Radio nuclear perfusion Surgery Ventilation/perfusion scan	11 1 2 1 1 2

Oesophagus	4	Dyspnea	4	100%	Autopsy	4

Pancreas	6	Dyspnea Fever None	4 1 2	83,30%	Autopsy EUS Ventilation/perfusion scan	5 1 2

Prostate	4	Dyspnea None	3 1	83,30%	Autopsy	4

Stomach	38	Cough Dyspnea Fever None	1 32 1 5	100%	Autopsy Pulmonary angiogram CT Aspiration cytology Ventilation/perfusion scan	37 1 2 1 3

Wilms' tumour	6	Abdominal pain Diffuse Dyspnea	1 4 2	83,30%	Autopsy CT Ventilation/perfusion scan	5 1 1

Others^*^	38	Chest pain Cough Dyspnea Fever None Shock	3 2 31 2 1 1	52,60%	Autopsy Pulmonary angiogram CT Aspiration cytology Radio nuclear perfusion EUS Surgery Ventilation/perfusion scan	16 2 19 1 4 1 12 5

^*^Others include bile duct, bone, leukemia, lymphoma, cartilage, cervix, choriocarcinoma, epidural, Ewing, fibroelastoma, glioblastoma, cardiac leiomyomatosis, multiple myeloma, ovary, parotid gland, sphenoid sinus, testis, thyroid, synovial sarcoma, unknown, and uterus.
