# Supplementary material for: Sudden Unexpected Death in a Patient with Tumour Associated Pulmonary Embolism
Source: Case Rep Med. 2014 Nov 27;2014:396832. doi: 10.1155/2014/396832 (PMC4265546; doi:10.1155/2014/396832)
Supplement: Supplementary file 1 — Overview of reported cases and their references. [file 396832.f1.pdf]

Supplementary Table 1: Overview of reported cases and their references (references listed on the next page)

Malignancy	Number of Cases	References
Atrial myxoma	9	[1-9]
Bladder	4	[10-13]
Breast	30	[14,15,10,16-29,13]
Colon	5	[30,31,25,32,13]
Kidney	10	[33-36,7,25,37-39]
Liver	9	[40,19,41-43,23,25,28,13]
Lung	12	[44,45,25,28,13]
Oesophagus	4	[46,28]
Pancreas	6	[18,47-49,28,13]
Prostate	4	[19,25,13]
Stomach	38	[15,50,51,19,52,25,28,13]
Wilm's tumour	6	[53-55]
Others	38	[56-63,17,64-79,7,25,28,80,81]

References

1. Doguet F, Hauville C, Godin M (2011) A biatrial myxoma revealed by pulmonary embolism. Archives of cardiovascular diseases 104 (6-7):421-422. doi:10.1016/j.acvd.2010.08.004
2. Fracasso T, Varchmin-Schultheiss K (2009) Sudden death due to pulmonary embolism from right atrial myxoma. International journal of legal medicine 123 (2):157-159. doi:10.1007/s00414-008-0312-9
3. Hein W, Kronert H, Falk V, Wittekind C, Hasenfuss G (2009) [A 17-year-old with cardiac mass and pulmonary embolism]. Der Internist 50 (12):1408-1414. doi:10.1007/s00108-009-2377-z
4. Liu Q, Zuo C, Lv T, Cui B (2013) Pulmonary embolism caused by right atrial myxoma on FDG PET/CT. Clinical nuclear medicine 38 (11):928-930. doi:10.1097/RLU.0000000000000226
5. Redford DT, Thompson JL, McCulloch JC, Nielsen VG (2014) Left atrial myxoma presenting as pulmonary embolism: potential role of heme oxygenase-1. Blood coagulation & fibrinolysis : an international journal in haemostasis and thrombosis. doi:10.1097/MBC.0000000000000097
6. Sato H, Tanaka T, Kasai K, Kita T, Tanaka N (2008) Sudden death due to acute pulmonary embolism from asymptomatic right atrial myxoma. Journal of forensic and legal medicine 15 (7):454-456. doi:10.1016/j.jflm.2008.02.013
7. Shepard JA, Moore EH, Templeton PA, McLoud TC (1993) Pulmonary intravascular tumor emboli: dilated and beaded peripheral pulmonary arteries at CT. Radiology 187 (3):797-801. doi:10.1148/radiology.187.3.8497633
8. Subban V, Lakshmanan A, Sethurathinam R, Ajit MS (2012) Right atrial myxoma--an unusual cause of pulmonary embolism. Journal of cardiac surgery 27 (5):604. doi:10.1111/j.1540-8191.2011.01414.x
9. Zhang C, Liu X, Ma G, Zhang H, Wang C, Liu J, Chen G, Li X, Miao Q (2012) Pulmonary embolization as the primary clinical manifestation of intravenous leiomyomatosis with intracardiac extension. The Annals of thoracic surgery 94 (3):1012. doi:10.1016/j.athoracsur.2012.02.019
10. Chakeres DW, Spiegel PK (1982) Fatal pulmonary hypertension secondary to intravascular metastatic tumor emboli. AJR American journal of roentgenology 139 (5):997-1000. doi:10.2214/ajr.139.5.997
11. Dhillon SS, Singh DJ, Dass B, Schaub CR (2001) Transitional cell carcinoma manifesting as acute cor pulmonale: cause of microscopic tumor embolism. Southern medical journal 94 (10):1030-1032

12. Fujiwara H, Katsura H, Suemur M (2006) [A case of pulmonary tumor embolism presenting with an initial manifestation of urothelial carcinoma]. Nihon Kokyuki Gakkai zasshi = the journal of the Japanese Respiratory Society 44 (7):504-510
13. von Herbay A, Illes A, Waldherr R, Otto HF (1990) Pulmonary tumor thrombotic microangiopathy with pulmonary hypertension. Cancer 66 (3):587-592
14. Abbondanzo SL, Klappenbach RS, Tsou E (1986) Tumor cell embolism to pulmonary alveolar capillaries. Cause of sudden cor pulmonale. Archives of pathology & laboratory medicine 110 (12):1197-1198
15. Babar SI, Sobonya RE, Snyder LS (1998) Pulmonary microvascular cytology for the diagnosis of pulmonary tumor embolism. The Western journal of medicine 168 (1):47-50
16. Chatkin JM, Fritscher LG, Fiterman J, Fritscher CC, da Silva VD (2007) Microscopic pulmonary neoplastic emboli: report of a case with respiratory failure but normal imaging. Primary care respiratory journal : journal of the General Practice Airways Group 16 (2):115-117. doi:10.3132/pcrj.2007.00020
17. Crane R, Rudd TG, Dail D (1984) Tumor microembolism: pulmonary perfusion pattern. Journal of nuclear medicine : official publication, Society of Nuclear Medicine 25 (8):877-880
18. Domanski MJ, Cunnion RE, Fernicola DJ, Roberts WC (1993) Fatal cor pulmonale caused by extensive tumor emboli in the small pulmonary arteries without emboli in the major pulmonary arteries or metastases in the pulmonary parenchyma. The American journal of cardiology 72 (2):233-234
19. Kane RD, Hawkins HK, Miller JA, Noce PS (1975) Microscopic pulmonary tumor emboli associated with dyspnea. Cancer 36 (4):1473-1482
20. Kridel R, Myit S, Pache JC, Gaspoz JM (2008) Pulmonary tumor embolism: a rare cause of acute right heart failure with elevated D-dimers. Journal of thoracic oncology : official publication of the International Association for the Study of Lung Cancer 3 (12):1482-1483. doi:10.1097/JTO.0b013e31818e107c
21. Moores LK, Burrell LM, Morse RW, Belgrave CH, Balingit AG (1997) Diffuse tumor microembolism: a rare cause of a high-probability perfusion scan. Chest 111 (4):1122-1125
22. Nakamura H, Adachi H, Sudoh A, Yagyu H, Kishi K, Oh-ishi S, Kusama H, Hashimoto T, Matsuoka T (2004) Subacute cor pulmonale due to tumor embolism. Internal medicine 43 (5):420-422
23. Schriner RW, Ryu JH, Edwards WD (1991) Microscopic pulmonary tumor embolism causing subacute cor pulmonale: a difficult antemortem diagnosis. Mayo Clinic proceedings 66 (2):143-148
24. Shapiro JM, Avigan De Fau - Warshofsky MK, Warshofsky Mk Fau - Greenebaum E, Greenebaum E Fau - Cole RP, Cole RP (1993) Acute cor pulmonale due to tumor emboli. Diagnosis by pulmonary artery catheterization. (0028-7628 (Print))

25. Shields DJ, Edwards WD (1992) Pulmonary hypertension attributable to neoplastic emboli: An autopsy study of 20 cases and a review of literature. Cardiovascular Pathology 1 (4):279-287. doi:http://dx.doi.org/10.1016/1054-8807(92)90038-P
26. Sostman HD, Brown M, Toole A, Bobrow S, Gottschalk A (1981) Perfusion scan in pulmonary vascular/lymphangitic carcinomatosis: the segmental contour pattern. AJR American journal of roentgenology 137 (5):1072-1074. doi:10.2214/ajr.137.5.1072
27. Uga S, Ikeda S, Matsukage S, Hamada M (2012) An autopsy case of acute cor pulmonale and paradoxical systemic embolism due to tumour cell microemboli in a patient with breast cancer. BMJ case reports 2012. doi:10.1136/bcr-2012-006682
28. Uruga H, Fujii T, Kurosaki A, Hanada S, Takaya H, Miyamoto A, Morokawa N, Homma S, Kishi K (2013) Pulmonary tumor thrombotic microangiopathy: a clinical analysis of 30 autopsy cases. Internal medicine 52 (12):1317-1323
29. Veinot JP, Ford Se Fau - Price RG, Price RG (1992) Subacute cor pulmonale due to tumor embolization. (0003-9985 (Print))
30. Heithaus RE, Jr., Hitchcock MA, Guileyardo JM (2013) Pulmonary tumor embolism syndrome from occult colonic adenocarcinoma. Proceedings 26 (3):290-292
31. Mihali E, Muresan M, Rusu ML, Fodor D (2013) Cardiac metastasis and tumor embolism in a patient with adenocarcinoma of the colon presenting with paraneoplastic polymyositis. Romanian journal of morphology and embryology = Revue roumaine de morphologie et embryologie 54 (3 Suppl):897-900
32. Uemura K, Nakajima M, Yamauchi N, Fukayama M, Yoshida K (2004) Sudden death of a patient with primary hypereosinophilia, colon tumours, and pulmonary emboli. Journal of clinical pathology 57 (5):541-543
33. Eggener SE, Dalton DP (2004) Bilateral pulmonary artery tumour emboli from renal carcinoma. The lancet oncology 5 (3):173. doi:10.1016/S1470-2045(04)01419-6
34. Garcia-Covarrubias L, Salerno TA, Robinson PG, Ciancio G (2008) Right atrial and pulmonary tumor embolism from renal rhabdomyosarcoma. Journal of cardiac surgery 23 (6):778-780. doi:10.1111/j.1540-8191.2008.00599.x
35. Li X, Li Q, Miao Y, Xu H, Liu Y, Qiu X, Wang EH (2013) A case of renal angiomyolipoma with intracardiac extension and asymptomatic pulmonary embolism. International journal of clinical and experimental pathology 6 (6):1180-1186
36. Morabito RA, Talug C, Zaslau S, Kandzari S (2010) Asymptomatic advanced pediatric papillary renal cell carcinoma presenting as a pulmonary embolus. Urology 76 (1):153-155. doi:10.1016/j.urology.2009.09.016
37. Shim H, Kim WS, Kim YW, Yang SS, Kim DK (2012) Successful management of pulmonary and inferior vena cava tumor embolism from renal cell carcinoma. The Korean journal of thoracic and cardiovascular surgery 45 (5):323-325. doi:10.5090/kjtcs.2012.45.5.323

38. Shimada S, Saito H, Arai Y (2012) Pulmonary embolism caused by spontaneous migration of tumor thrombus of renal cell carcinoma: a report of two cases. International journal of urology : official journal of the Japanese Urological Association 19 (3):277-278. doi:10.1111/j.1442-2042.2011.02926.x
39. Taylor JS, Breau RH, Cagiannos I, Morash C (2007) Renal sarcoma and associated malignant pulmonary embolism: a report of 2 cases. Canadian Urological Association journal = Journal de l'Association des urologues du Canada 1 (2):123-125
40. DeVita VT, Trujillo NP, Blackman AH, Ticktin HE (1965) Pulmonary manifestations of primary hepatic carcinoma. The American journal of the medical sciences 250 (4):428-436
41. Lin HH, Hsieh CB, Chu HC, Chang WK, Chao YC, Hsieh TY (2007) Acute pulmonary embolism as the first manifestation of hepatocellular carcinoma complicated with tumor thrombi in the inferior vena cava: surgery or not? Digestive diseases and sciences 52 (6):1554-1557. doi:10.1007/s10620-006-9129-x
42. Nakanishi M, Hige S, Chuma M, Asaka M (2006) Pulmonary tumor embolism in a case of hepatocellular carcinoma. Journal of gastroenterology 41 (8):808-809. doi:10.1007/s00535-006-1850-x
43. Papp E, Keszthelyi Z, Kalmar NK, Papp L, Weninger C, Tornoczky T, Kalman E, Toth K, Habon T (2005) Pulmonary embolization as primary manifestation of hepatocellular carcinoma with intracardiac penetration: a case report. World journal of gastroenterology : WJG 11 (15):2357-2359
44. Chen WL, Cherng SC, Hwang WS, Wang DJ, Wei J (1991) Perfusion scan in pulmonary tumor microembolism: report of a case. Journal of the Formosan Medical Association = Taiwan yi zhi 90 (9):863-866
45. Liang YH, Kuo SW, Lin YL, Chang YL (2011) Disseminated microvascular pulmonary tumor embolism from non-small cell lung cancer leading to pulmonary hypertension followed by sudden cardiac arrest. Lung cancer 72 (1):132-135. doi:10.1016/j.lungcan.2010.12.022
46. Soares FA, Landell GA, de Oliveira JA (1991) Pulmonary tumour embolism from squamous cell carcinoma of the oesophagus. European journal of cancer 27 (4):495-498
47. Sancho-Chust JN, Ferreres J Fau - Pineda J, Pineda J Fau - Molla MA, Molla Ma Fau - Giner F, Giner F Fau - Juan M, Juan M Fau - Blanquer J, Blanquer J (2009) Pulmonary tumor embolism as an initial manifestation of pancreatic adenocarcinoma. (0020-1324 (Print))
48. Steiner S, Plehn G, Reinecke P, Cohnen M, Schwartzkopff B, Hennersdorf MG, Strauer BE (2004) Disseminated microvascular pulmonary tumor cell embolism: a rare cause of fulminant pulmonary hypertension. Onkologie 27 (6):566-568. doi:10.1159/000081340
49. Tee CT, Chen HC, Segarajasingam DS, Yusoff IF (2013) Pulmonary embolism diagnosed with EUS on a patient with adenocarcinoma of the pancreas (with video). Gastrointestinal endoscopy 78 (3):542-543; discussion 543. doi:10.1016/j.gie.2013.05.001

50. Cheung TC, Ng FH, Chow KC, Maw CK, Ng WF (1997) Occult gastric cancer presenting as cor pulmonale resulting from tumor cell microembolism. The American journal of gastroenterology 92 (6):1057-1059
51. Iwakami S, Sato T, Takagi H, Fujii M, Iwakami N, Yoshimi K, Koyama R, Ichikawa M, Yoshioka M, Takahashi K (2009) An autopsy case of subacute cor pulmonale due to pulmonary tumor cell emboli in a patient with gastric cancer. Internal medicine 48 (12):1057-1060
52. Shalev T, Guranda L, Shovman O, Michael L (2004) Microscopic pulmonary tumor embolism in adenocarcinoma of the stomach. The Israel Medical Association journal : IMAJ 6 (6):374-375
53. Li L, Light J, Marchick M, Hoelle R (2014) Dyspnea, tachycardia, and new onset seizure as a presentation of wilms tumor: a case report. Case reports in emergency medicine 2014:562672. doi:10.1155/2014/562672
54. van den Heuvel-Eibrink MM, Lankhorst B Fau - Egeler RM, Egeler Rm Fau - Corel LJA, Corel Lj Fau - Kollen WJW, Kollen WJ (2008) Sudden death due to pulmonary embolism as presenting symptom of renal tumors. (1545-5017 (Electronic))
55. Zakowski MF, Edwards RH, McDonough ET (1990) Wilms' tumor presenting as sudden death due to tumor embolism. Archives of pathology & laboratory medicine 114 (6):605-608
56. Ban D, Yamamoto S, Kuno H, Fujimoto H, Fujita S, Akasu T, Moriya Y (2008) A case of huge colon carcinoma and right renal angiomyolipoma accompanied by proximal deep venous thrombosis, pulmonary embolism and tumor thrombus in the renal vein. Japanese journal of clinical oncology 38 (10):710-714. doi:10.1093/jjco/hyn094
57. Begin LR, Raptis S (1991) Diffuse pulmonary carcinomatous embolization: a rare and fatal manifestation of ovarian cancer. Gynecologic oncology 41 (3):250-254
58. Berber I, Bentli R, Erkurt MA, Ulutas O, Ediz C, Nizam I, Kirici Berber N, Unlu S, Koroglu R, Koroglu M, Akpolat N (2014) Pulmonary embolism as the initial presentation of testicular carcinoma. (2090-6706 (Print)). doi:D - NLM: PMC3872100 EDAT- 2014/01/03 06:00 MHDA- 2014/01/03 06:01 CRDT- 2014/01/03 06:00 PHST- 2013/10/03 [received] PHST- 2013/11/13 [accepted] PHST- 2013/12/09 [epublish] AID - 10.1155/2013/264569 [doi] PST - ppublish
59. Bhuvaneswaran JS, Venkitachalam CG, Sandhyamani S (1993) Pulmonary wedge aspiration cytology in the diagnosis of recurrent tumour embolism causing pulmonary arterial hypertension. International journal of cardiology 39 (3):209-212
60. Blanc AL, Jardin C, Faivre JB, Le Rouzic O, Do Cao C, Benhamed L, Ramon PP, Fournier C (2011) Pulmonary artery tumour-embolism diagnosed by endobronchial ultrasound-guided transbronchial needle aspiration. The European respiratory journal 38 (2):477-479. doi:10.1183/09031936.00182210

61. Bozaci EA, Taskin S, Gurkan O, Atasoy C, Ersoy ZG, Erekul S, Numanoglu N, Ortac F (2005) Intracavitary cardiac metastasis and pulmonary tumor emboli of choriocarcinoma: the first case diagnosed and treated without surgical intervention. Gynecologic oncology 99 (3):753-756. doi:10.1016/j.ygyno.2005.07.012
62. Buderi S, Theologou T, Gosney J, Shackcloth M (2013) Pulmonary artery tumor embolism in a patient with previous fibroblastic osteosarcoma. The Annals of thoracic surgery 95 (6):2155-2157. doi:10.1016/j.athoracsur.2012.10.062
63. Burgoyne LL, Anghelescu DL, Tamburro RF, De Armendi AJ (2006) A pediatric patient with a mediastinal mass and pulmonary embolus. Paediatric anaesthesia 16 (4):487-491. doi:10.1111/j.1460-9592.2005.01765.x
64. Han S, Chaya C, Hoo GW (2006) Thrombolytic therapy for massive pulmonary embolism in a patient with a known intracranial tumor. Journal of intensive care medicine 21 (4):240-245. doi:10.1177/0885066606287047
65. Hibino M, Akazawa K, Hikino K, Oe M (2012) Pulmonary tumor embolism secondary to uterine corpus carcinosarcoma mimicking pulmonary thromboembolism. Internal medicine 51 (18):2603-2607
66. Huang CY, Huang CH, Yang AH, Wu MH, Ding YA, Yu WC (2003) Solitary pulmonary artery myxoma manifesting as pulmonary embolism and subacute cor pulmonale. The American journal of medicine 115 (8):680-681
67. Kim DH, Shim JK, Choi YS, Kwak YL (2009) Distinct capnographic waveform in a pulmonary embolism caused by intravenous leiomyomatosis. Anaesthesia 64 (4):453-455. doi:10.1111/j.1365-2044.2009.05907.x
68. Ko YM, Lee SH, Huh J, Koo HH, Yang JH (2012) A fatal case of acute pulmonary embolism caused by right ventricular masses of acute lymphoblastic lymphoma-leukemia in a 13 year old girl. Korean journal of pediatrics 55 (7):249-253. doi:10.3345/kjp.2012.55.7.249
69. Lambert-Jensen P, Mertz H Fau - Nyvad O, Nyvad O Fau - Christensen JH, Christensen JH (1994) Subacute cor pulmonale due to microscopic pulmonary tumour cell embolization. (0954-6820 (Print))
70. Mansencal N, El Hajjam M, Vieillard-Baron A, Pelage JP, Lacombe P, Dubourg O (2006) Recurrent pulmonary embolism with non-mobile thrombus in a patient with leiomyosarcoma of the left renal vein. International journal of cardiology 112 (2):247-248. doi:10.1016/j.ijcard.2005.07.044
71. Morgan JA, Paone G (2013) Chondrosarcoma presenting as a saddle tumor pulmonary embolism. (1540-8191 (Electronic))
72. Mutlu GM, Factor P (2006) Pulmonary tumor embolism of unknown origin. Mayo Clinic proceedings 81 (6):721. doi:10.4065/81.6.721
73. Odeh M, Oliven A, Misselevitch I, Boss JH (1997) Acute cor pulmonale due to tumor cell microemboli. Respiration; international review of thoracic diseases 64 (5):384-387

74. Perez Baztarrica G, Nieva N, Gariglio L, Salvaggio F, Porcile R (2010) Images in cardiovascular medicine. Primary cardiac lymphoma: a rare case of pulmonary tumor embolism. Circulation 121 (20):2249-2250. doi:10.1161/CIRCULATIONAHA.109.863126
75. Pinder MA, Dibardino DJ, Collier AB, Knudson JD (2014) Chondrosarcoma presenting with pulmonary embolism in a 9-year-old girl: a case report. Cardiology in the young:1-4. doi:10.1017/S1047951114000274
76. Rekik S, Hentati M, Boudawara T, Abdennadher M, Frikha I, Kammoun S (2009) Myofibroblastic tumor of the right ventricle causing bilateral pulmonary embolism in a 31 year-old woman. International journal of cardiology 131 (3):e131-133. doi:10.1016/j.ijcard.2007.07.082
77. Ribeiro V, Almeida J, Madureira AJ, Lopez E, Machado L, Albuquerque R, Pinho P (2013) Intracardiac leiomyomatosis complicated by pulmonary embolism: a multimodality imaging case of a rare entity. The Canadian journal of cardiology 29 (12):1743 e1741-1743. doi:10.1016/j.cjca.2013.09.008
78. Schoneveld JM, Debing E, Verfaillie G, Geers C, Van den Brande P (2012) Deep venous thrombosis and pulmonary embolism caused by an intravascular synovial sarcoma of the common femoral vein. Vascular and endovascular surgery 46 (8):693-695. doi:10.1177/1538574412460770
79. Shah RA, Kalidoss L, Mohanraj A, Lakshmanan A, Thirumurthi MK, Ajit MS (2014) Papillary fibroelastoma of tricuspid valve presenting as pulmonary embolism. Asian cardiovascular & thoracic annals. doi:10.1177/0218492314526601
80. Varma JS (1982) Subacute pulmonary hypertension and systemic tumour embolism from cervical cancer. Scottish medical journal 27 (4):336-340
81. Villa D, Knowling M (2010) Acute saddle pulmonary embolism in Ewing sarcoma. Journal of pediatric hematology/oncology 32 (5):e210-212. doi:10.1097/MPH.0b013e3181dce311
